# Bilateral Cataract After Electric Injury in a Child: A Case Report

**DOI:** 10.7759/cureus.110722

**Published:** 2026-06-12

**Authors:** Chandrashekhar B Kale, Gargi Wavikar, Chandrashekhar M Wavikar, Mamta N Tanna, Maninder S Setia

**Affiliations:** 1 Ophthalmology, Wavikar Eye Hospital/Wavikar Eye Institute, Thane, IND; 2 Epidemiology, Wavikar Eye Hospital/Wavikar Eye Institute, Thane, IND

**Keywords:** child, electric cataracts, electric injuries, ophthalmologic manifestations, surgery, vision

## Abstract

Electric injuries constitute an important group of injuries, and the number of cases has increased in recent years. We present a case of bilateral cataract in a child following an electrical injury. A 12-year-old male child presented to our ophthalmology center with a progressive reduction in vision in both eyes over the preceding month. The patient reported experiencing an electric shock one year earlier from a live wire carrying approximately 220 V (based on the available history, although this could not be confirmed). The child sustained injuries to the body and head, for which he was hospitalized and managed at a private hospital for 18 days. On examination, the child had large hyperpigmented and depigmented healed thermal burn scars over the lateral abdomen and back, along with areas of scarring alopecia on the scalp. The uncorrected distance visual acuity (UDVA) in the right eye was 6/60, improving to 6/18 with a pinhole on the Snellen visual acuity chart. In the left eye, the UDVA was also 6/60 and improved to 6/12 with a pinhole. The corrected near visual acuity (CNVA) was N18 on the Snellen near vision chart in both eyes. Slit-lamp examination revealed Grade 3 anterior cortical cataracts in both eyes. No other ophthalmological abnormalities were detected. The child underwent bilateral cataract surgery under general anesthesia. A hydrophobic foldable posterior chamber monofocal intraocular lens was implanted in the capsular bag following phacoemulsification. Surgery in both eyes was uneventful, and no postoperative complications were observed. Both distance and near vision improved after surgery. We present this case of electrical cataract, a relatively uncommon but well-documented complication of electrical burn injury, to highlight the occurrence of this condition following electrical injuries. Healthcare professionals managing such patients should be aware of these complications, and patients should be informed and counselled regarding the possible ocular sequelae, some of which may develop years after the initial injury. Therefore, patients with electrical injuries should undergo regular ophthalmological examinations to facilitate early identification of these complications. Appropriate management can lead to significant visual improvement, as demonstrated in our case.

## Introduction

Electric injuries constitute an important group of injuries, and the number of cases has increased in recent years [1]. Epidemiological studies from India have shown that the majority of cases occur in males [2-4]. In addition, studies have reported cases across a wide age range, from 1 to 70 years, although a high proportion of cases occur in the 21-30-year age group [2-4]. These injuries are classified as low-voltage when caused by currents of <1000 V and high-voltage when caused by currents of >1000 V. A review reported that approximately 38% of electrical injuries were high-voltage injuries, while the voltage was not specified in 43.7% of cases [5,6]. Although most of these incidents in adults may be occupationally related, hospital admissions due to electrical injuries and burns have also been reported in children [7-9]. Furthermore, studies have shown that a large proportion of electrical injuries in children are caused by low-voltage currents, including those below 100 V [10].

The most common clinical manifestations of electrical injuries include thermal burns, pain, tissue damage, loss of consciousness, respiratory arrest, fractures, and intra-abdominal trauma [5,6]. Initial management may involve cardiac and respiratory stabilization in the emergency department, along with treatment of associated conditions such as burns, fractures, and hemodynamic instability [5]. One study reported that 82% of patients with electrical injuries experienced long-term consequences, the most common being neurological and psychological sequelae [11]. The eye is another important organ that may be affected by electrical injuries. Ocular manifestations can involve various structures, including the eyelids, conjunctiva (chemosis), cornea (edema and related complications), uvea (anterior uveitis), retina, and lens (cataract formation) [12]. These conditions may require specialized ophthalmological care. Cataracts occurring after electrical injuries have been referred to in the literature as electrical cataracts [13-15]. We present a case of bilateral cataract in a child following an electrical injury.

## Case presentation

**Figure 1:**
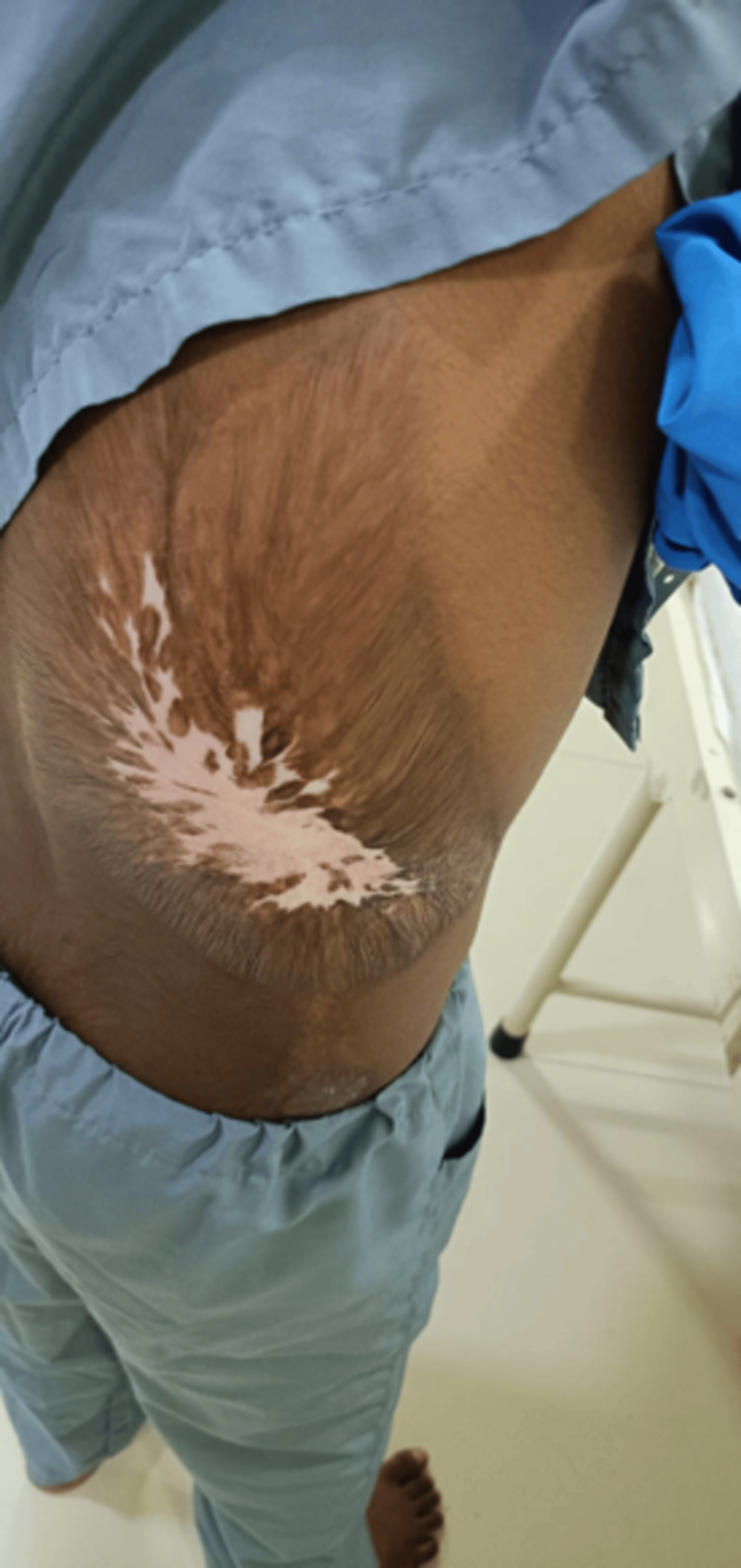
Hyperpigmented and depigmented healed thermal burn scar on the lateral abdomen and back

**Figure 2:**
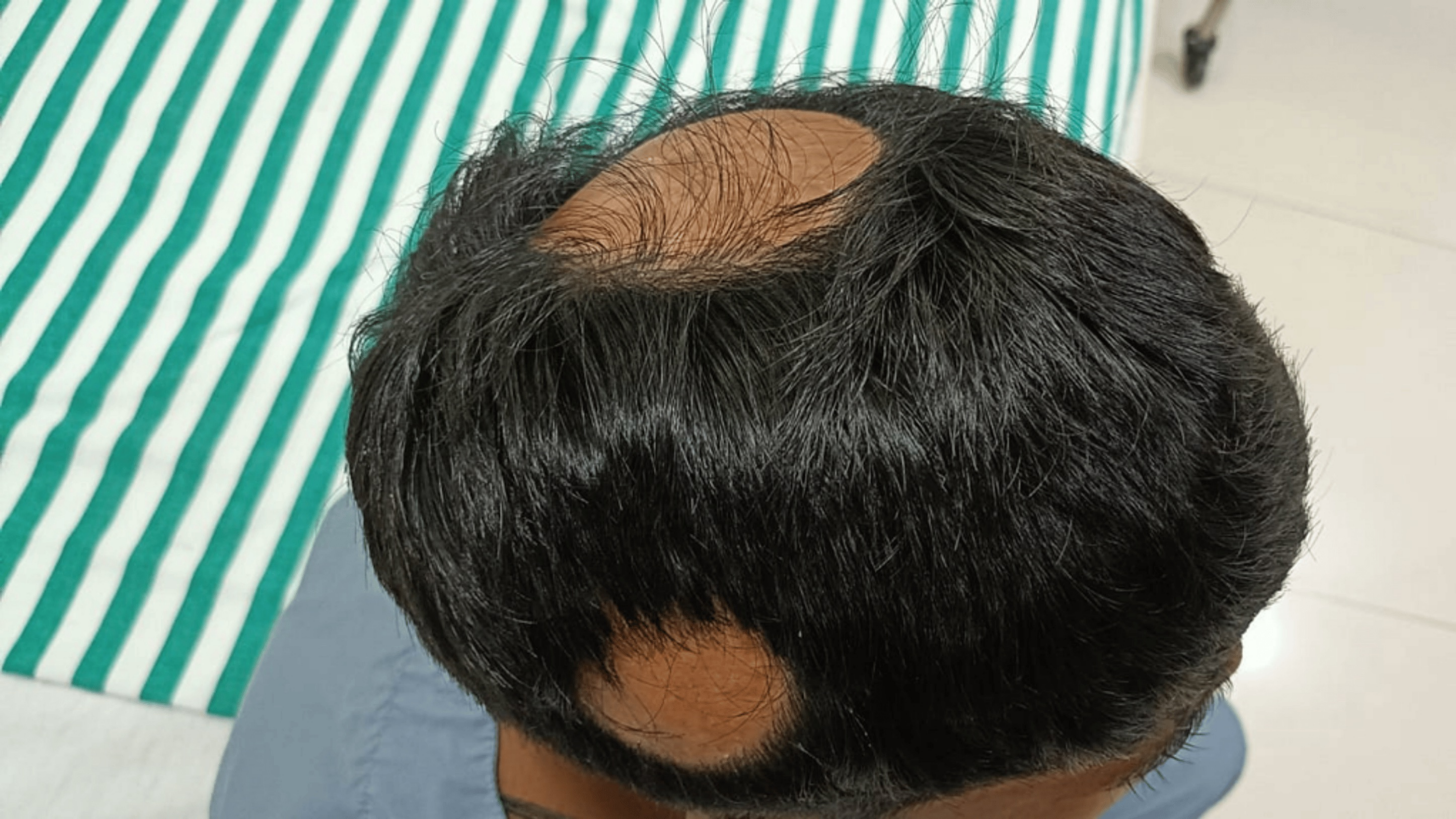
Two areas of scarring alopecia on the scalp following an electrical injury

We present the case of a 12-year-old male child who presented to our ophthalmology center with a progressive reduction in vision in both eyes over the preceding month. The patient reported experiencing an electric shock one year earlier from a live wire carrying approximately 220 V (based on the available history, although this could not be confirmed). The child sustained injuries to the body and head, for which he was hospitalized and managed at a private hospital for 18 days. On examination, we observed a large hyperpigmented and depigmented healed thermal burn scar over the lateral abdomen and back (Figure 1). In addition, the child had areas of scarring alopecia on the scalp (Figure 2). There were no active wounds on the body. The child was not examined by an ophthalmologist during his hospital stay and was not advised to undergo ophthalmological follow-up at the time of discharge. However, he subsequently presented to our center with complaints of progressive visual impairment.

**Figure 3:**
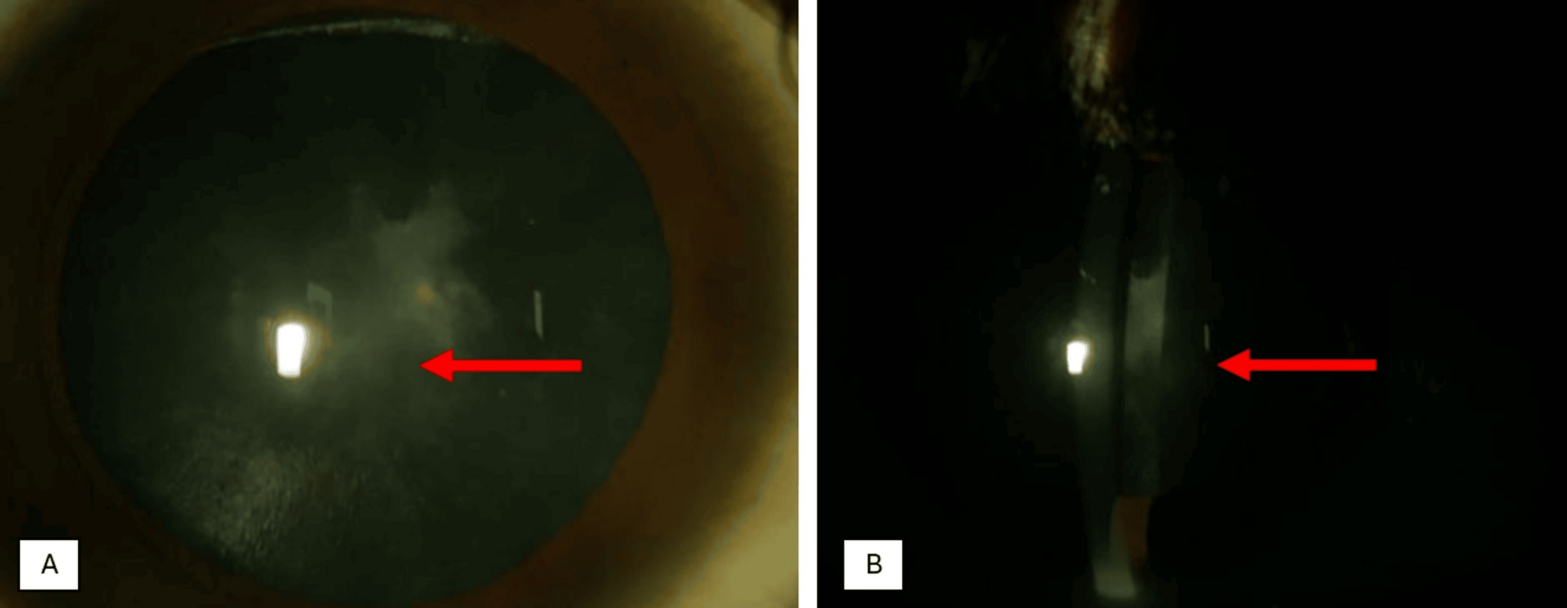
Dense anterior cortical cataract in the right eye (A) and left eye (B) The arrow points to the cortical cataract.

On ophthalmic examination, the external eye was normal, and no injury marks were seen on the eyelids or periocular region. The uncorrected distance visual acuity (UDVA) was 6/60 in the right eye, improving to 6/18 with a pinhole on the Snellen visual acuity chart. In the left eye, the UDVA was also 6/60, improving to 6/12 with a pinhole. The best corrected near visual acuity (CNVA) was N18 on the Snellen near vision chart in both eyes. Slit-lamp examination revealed a Grade 3 anterior cortical cataract in both eyes, with no polychromatic luster observed (Figure 3). There was no lid or conjunctival hyperemia or congestion. We also did not observe any corneal epithelial necrosis, corneal opacities, synechiae formation, or iritis. The pupils were normal in size and reactivity, and no pupillary spasm was observed. The posterior segment was examined after pharmacological dilation using a 90 D lens at the slit lamp and a 20 D indirect ophthalmoscope. No evidence of optic neuritis, retinal edema, retinal hemorrhages, macular holes, retinal detachment, or papilloedema was observed. There was no photophobia or blepharospasm. Based on these findings, a diagnosis of bilateral electrical cataract was made.

The child underwent surgery for bilateral cataracts. The right eye was operated first on under general anesthesia. A hydrophobic foldable posterior chamber monofocal intraocular lens was implanted in the capsular bag following phacoemulsification cataract surgery. The left eye was operated on after 15 days, using the same surgical procedure and intraocular lens. Both surgeries were uneventful, and no postoperative complications were observed. At one week postoperatively, UDVA improved to 6/6 on the Snellen visual acuity chart in both eyes. CNVA improved to N6 on the Snellen near vision chart. The patient did not report any complications up to the last follow-up visit at our center.

## Discussion

We present this case of electrical cataract, a relatively uncommon but well-documented complication of electrical burn injury. The patient was a 12-year-old child who developed visual symptoms approximately one year after the injury. The patient underwent surgery and achieved good visual recovery, with no major complications reported up to the last follow-up visit at our center.

Ocular changes following electrical injuries have been reported by several authors. A review on ophthalmological manifestations of electrical and lightning trauma reported cataracts in approximately 30% of cases, with a higher association in high-voltage injuries [16]. Case reports by Hashemi et al. and Reddy, along with literature reviews, have suggested that the prevalence of cataract formation ranges from less than 1% to as high as 8% [15,17,18]. Saffle and co-workers studied 113 patients with electrical injuries and reported an incidence of 6.2% [19]. In our case, the patient was a 12-year-old child with a reported exposure to 220 V electricity. Although previous studies have mainly reported cataracts following high-voltage injuries (>10,000 V), several cases associated with low-voltage exposure have also been described, similar to our case [15,17,20-23].

Our patient had healed scars over the scalp, abdomen, and back, indicating that electrical burn injuries involved multiple body regions, including the scalp. It has been suggested that current passing through the head, closer to the ocular structures, may result in ocular burns and cataract formation [15,18,24]. Therefore, healthcare professionals managing electrical injuries should be aware that patients with scalp involvement should be counselled regarding the risk of delayed cataract formation. Regular ophthalmological follow-up is essential for early detection and timely intervention. Although bilateral cataracts are more commonly reported, rare cases of unilateral involvement have also been described in the literature [13,25-27]. Piedrahita et al., in a review, reported that posterior subcapsular cataracts were most common, followed by anterior subcapsular cataracts, while approximately 10% were cortical cataracts [16]. In our case, the patient had bilateral anterior cortical cataracts. The latency period between electrical injury and the onset of visual symptoms was approximately 12 months. Previous reports have described latency periods ranging from three months to as long as eight years [14,28], with an exceptionally rare case reported at 27 years. This highlights the need for long-term follow-up, as ocular complications of electrical injuries may manifest even years later. Although associated ocular pathologies such as uveitis, retinal detachment, and macular cysts have been reported in the literature, our patient did not demonstrate any additional ophthalmic abnormalities [16,20]. This likely contributed to the good visual outcome after surgery, as the absence of other ocular pathology is associated with a better prognosis [17]. A detailed fundus and neurological examination is therefore essential in such patients.

## Conclusions

The present case highlights the occurrence of this complication following electrical injuries. Healthcare professionals managing such patients should be aware of these complications, and patients should be informed and counselled regarding possible ocular sequelae, some of which may appear years after the initial injury. Therefore, patients with electrical injuries should undergo regular ophthalmological examinations to detect these complications early. Appropriate management can lead to significant visual improvement, as demonstrated in our case.
